# The Use of Premixed Drugs in Commodity Packets in the Population: Prevalence and Correlates Revealed by the 2018 National Survey of Substance Use in Taiwan

**DOI:** 10.2188/jea.JE20220356

**Published:** 2024-05-05

**Authors:** Shang-Chi Wu, Lian-Yu Chen, Po-Chang Hsiao, Te-Tien Ting, Cheng-Fang Yen, Shu-Sen Chang, Chung-Yi Li, Hao-Jan Yang, Chia-Feng Yen, Chuan-Yu Chen, Jiun-Hau Huang, Yu-Kang Tu, Wei J. Chen

**Affiliations:** 1 Institute of Epidemiology and Preventive Medicine, College of Public Health, National Taiwan University, Taipei, Taiwan; 2 Department of Addiction Psychiatry and Kunming Prevention and Control Center, Taipei City Hospital, Taipei, Taiwan; 3 Department of Forensic Medicine, School of Medicine, Taipei, Taiwan; 4 School of Big Data Management, Soochow University, Taipei, Taiwan; 5 Department of Psychiatry, Kaohsiung Medical University Hospital & School of Medicine and Graduate Institute of Medicine, College of Medicine, Kaohsiung Medical University, Kaohsiung, Taiwan; 6 Department of Public Health, College of Public Health, National Taiwan University, Taipei, Taiwan; 7 Institute of Health Behaviors and Community Sciences, College of Public Health, National Taiwan University, Taipei, Taiwan; 8 Department of Public Health, College of Medicine, National Cheng Kung University, Tainan, Taiwan; 9 Department of Public Health, College of Health Care and Management, Chung Shan Medical University, Taichung, Taiwan; 10 Department of Public Health, College of Medicine, Tzu Chi University, Hualien, Taiwan; 11 Institute of Public Health, National Yang-Ming Chiao Tung University, Taipei, Taiwan; 12 Center for Neuropsychiatric Research, National Health Research Institutes, Miaoli, Taiwan; 13 Department of Psychiatry, College of Medicine and National Taiwan University Hospital, National Taiwan University, Taipei, Taiwan

**Keywords:** drugs in drink packets, novel psychoactive substances, recreational use, illicit drug, national survey

## Abstract

**Background:**

Administering premixed drugs in commodity packets was first reported in Asia in 2015, but there continues to be a dearth of related population-based data. This study aimed at examining (1) the prevalence of drug-packet use in the population and (2) the sociodemographic profiles, particularly gender distribution, of drug-packet users.

**Methods:**

Data were derived from a survey of 18,626 Taiwanese civilians, aged 12–64 years, using stratified, multi-stage, random sampling in 2018. Participants anonymously completed a computer-assisted self-interview on tablet computers which covered the use and problematic use of illicit drugs/inhalants, prescription drugs, and other psychoactive substances.

**Results:**

Approximately 1.46% of respondents had a lifetime use of illicit drugs, with drugs in commodity packets (0.18%) being ranked the fifth-most commonly used illicit drugs, higher than nitrous oxide (0.14%) and heroin (0.09%). Ten formats of drug packets were endorsed by users. Approximately 81.6% of persons with drug packet use had lifetime use of other illicit drugs. The correlates of the use of drugs in commodity packets were different from those of the exclusive use of other drugs, particularly concerning the lack of gender differences in the former category in the whole sample and the subgroups of various sociodemographic characteristics and other substance use.

**Conclusion:**

Drugs in commodity packets have become a common way of administering illicit drugs in the population in Taiwan, and there were no gender differences among users. Our findings have implications for more efficient drug testing and culturally appropriate intervention for drug-packet use.

## INTRODUCTION

A growing concern related to the global trend of illicit drug use is the emergence of novel psychoactive substances (NPSs), which are a diverse group of substances often known as either designer or synthetic drugs.^1^^–^^3^ In 2015, the Asian media started to report official warnings about adulterated instant beverage powders that were laced with illicit drugs in Taiwan^4^ and the presence of illicit drugs in premixed instant coffee seized in Malaysia.^5^ Case reports of acute toxicity and fatalities due to NPSs since 2016 have mentioned various drink packets and stamp bags laced with illicit drugs in many countries, including the United Kingdom,^6^ Japan,^7^ the United States,^8^ Singapore,^9^ and Taiwan.^10^^,^^11^ The finding that users of NPSs tended to be unaware of the contents of the compounds consumed was highlighted by the 2017 World Drug Report.^12^ This untraditional way of administering NPSs in the form of drugs in commodity packets, along with users remaining unaware of their contents, poses a challenge for regular drug surveys. Although national surveys in the United States,^13^ the European Union,^14^ and Japan^15^ inquired about specific categories of NPSs, a lack of population-based data on the use of drugs in commodity packets and its correlates remains.

Another issue concerning the global trend of illicit drug use is the diminishing gender difference. While most epidemiological data showed that the majority of drug users were males, females seem to use certain categories of drugs nearly as much as males do, especially NPSs.^16^ For instance, compared to other traditional types of drugs, minor gender differences in the use prevalence of NPSs were seen in recent surveys among the general population in Japan^15^ and university students in the Netherlands.^17^ Whether there is less of a gender difference in the use of drugs in commodity packets in the population or sociodemographic subgroups warrants further investigation.

To address the concern about the unorthodox delivery method of NPSs, the 2018 National Survey of Substance Use in Taiwan listed drugs in commodity packets as a new category of illicit premixed drugs in a variety of appealing packets for the first time in a national survey. Based on the 2018 national survey, this study aimed to examine (1) the prevalence of drug use in commodity packets in the population compared to that of other illicit drug use and (2) the sociodemographic profiles, particularly gender distribution, of persons who consume drugs in commodity packets.

## METHODS

### Participants

The National Survey of Substance Use is a national household survey that targets 12- to 64-year-old, noninstitutionalized individuals in the Taiwanese general population. Using a stratified, multistage, probability-proportional-to-size random sampling from the Taiwanese population household registry, 28,840 individuals were selected as potential participants in the 2018 survey. Finally, a total of 18,626 selected individuals agreed to complete a computer-assisted self-interview, corresponding to a response rate of 64.6%. The study was approved by the Research Ethics Committee of the National Taiwan University Hospital (NTUH-REC no. 201802031RINB).

### Survey methods

#### Stratification of the nation

To determine the stratification, we created an index using cluster analysis at the township level following previous studies^18^^,^^19^ with updated parameters: (1) population density (people/km^2^)^20^; (2) percentage of people with college or above educational levels^20^; (3) percentage of elderly people over 65 years old^20^; (4) percentage of people of agriculture workers^21^; and (5) the number of physicians per 100,000 people.^22^ Each county or metropolitan city was divided into one to three strata according to the clustering index, and the nation’s 358 townships/cities/districts were regrouped into 50 strata.

#### Sampling processes

Depending on the urbanity of each stratum, there were three types of sampling processes in this study: (1) using the neighborhood (*lin*) as the primary sampling unit (number of strata = 13), then selected individuals; (2) using the village (*chun-li*) in a city as the primary sampling unit (number of strata = 12), then selected neighborhoods and finally selected individuals; and (3) using the township/city/district (*hsiang-chen-shih-chu*) as the primary sampling unit (number of strata = 24), then selected neighborhoods and finally selected individuals. To increase the number of adolescent participants, one extra individual aged 12 to 17 living in the household of a selected participant was also invited to participate in the survey. The sampling size for each of 20 administrative units (14 counties and 6 special municipalities) ranged from approximately 1,100 to 2,600, with a final sampling size of 28,840.

#### Completion rate of the survey

Because of the completeness of household registration, stratified, multistage, probability proportional to size, random sampling could be executed. In addition, the completion rate of each township/city/district was incorporated into the final weight. By the end of the study, the response rate of completing the interview in each county or special municipality ranged from 30.9% in Hsinchu County to 91.3% in Tainan City (eTable 1).

#### Execution of the fieldwork

The tablet-based, computer-assisted self-interview (CASI) technology further allowed for many novel ways to carry out the fieldwork. First, each tablet linked via the internet to the personal information (ie, name and address) of the sampled participants allocated by the headquarters via the internet. Second, each tablet was equipped with a global positioning system that allowed the headquarters to track the location of each interview. Third, the encrypted data of the questionnaire completed by each participant could be transmitted back to a server at the headquarters.

During the fieldwork, the four regional centers were responsible for coordinating the administrative work and the evaluation, including monitoring the progress of the field workers, carrying out phone-based validation checks, and holding monthly meetings to ensure progress and data quality. Over 160 trained professional interviewers made household visits and approached the selected individuals. Several efforts were made to ensure the quality of the fieldwork. First, for those field workers who had completed at least 20 respondents, assistants at the headquarters made phone calls to the participants to ensure that on a specified day they were interviewed by the designated field worker. Among 119 field workers eligible for auditing, 99 field workers (83%) were audited for their fieldwork. Out of 601 participants who were contacted by the validation phone calls, 3 (0.5%) were found to be answered by other family members for the interview; hence, their data were deleted. Second, the global positioning system installed in the tablet recorded the time and location whenever a field worker turned on the tablet-based interview, which was then used to check against the sampling list for any mismatch. None of the field workers were found to have unreasonable clustering of the time-place global positioning system information. These checks led to the discarding of problematic data and indicated that the whole fieldwork was of good quality.

### Measurements

The CASI was implemented on touchscreen tablet computers. The questionnaire primarily consisted of sections regarding the use and problematic use of illicit drugs/inhalants, prescription drugs, and other psychoactive substances, as well as the participants’ sociodemographic and psychosocial correlates, among other information. Each participant was first asked whether he or she had ever used any of the individual categories of illicit drugs/inhalants listed in the questionnaire. Those who reported having used drug(s) were then asked for the time of the most recent use. As a newly enlisted category of drug, prefixed drug in commodity packets was described as “repacked addictive substance” accompanied by pictures of seized illicit drug packets in the questionnaire.

In addition, the degree of nicotine dependence was assessed using the 6-item Fagerström Test for Nicotine Dependence (FTND),^23^ with a cutoff score of 4 validated in male Taiwanese smokers.^24^ Alcohol use problems were examined using the Alcohol Use Disorders Identification Test (AUDIT),^25^ in which three strata (ie, 0–7, 8–13, and 14 or more) were derived from a stratum-specific likelihood ratio test among inpatients of a general hospital in Taipei.^26^ Problematic drug use was measured using the 20-item Drug Abuse Screening Test (DAST),^27^ and its validity has been demonstrated in psychiatric outpatients.^28^^,^^29^ One item in the DAST asking whether the participant has interpersonal problem with his/her parents or partners over the drug use was further used as an individual item in subsequent analyses.

In a pilot study of the validity and reliability of the questionnaire, 30 treatment-seeking patients at Taipei Psychiatric Center were recruited to be administered the CASI. Among those with a positive urinalysis result, the proportion of positive self-reporting (ie, sensitivity) was 24/25 (96%) for methamphetamine, 6/7 (86%) for marijuana, and 11/11 (100%) for ecstasy. Meanwhile, among those with a negative urinalysis result, the proportion of negative self-reporting (ie, specificity) was 5/5 (100%) for methamphetamine, 16/23 (69.6%) for marijuana, and 14/19 (73.7%) for ecstasy; a likely reason for self-reported drug use not detected in urinalysis was due to the detection time window. Furthermore, 16 of these patients and 15 participants recruited from high-risk community residents completed the CASI twice, 1 to 4 weeks apart. The consistency of their responses to questions about drug use ranged from 90% to 100% (eTable 2).

### Statistical analysis

In the estimation of the prevalence of illicit drug use, population- and response-rate-adjusted weights were given to each group classified by sex, age, and strata. Correlates of the use of drugs in commodity packets were examined using multivariable logistic regression with adjustment for potential confounding in sociodemographic characteristics. All statistical analyses were performed using SAS 9.4 software (SAS Institute Inc., Cary, NC, USA) via methods that can address the complex survey design. Hence, the distribution of characteristics and cross-tabulations were completed using PROC SURVEYFREQ and PROC SURVEYMEANS, while multivariable logistic regression analyses were performed using PROC SURVEYLOGISTIC.

## RESULTS

The distributions of the demographic characteristics in the samples of the 2018 survey were similar to those of the counterparts in the nation (Table 1).

**Table 1.** Characteristics of the participants in the 2018 National Survey of Substance Use in Taiwan and comparison with the corresponding general population in the same year

Variables	2018 Survey Participants (*N* = 18,626)			General population^a^ (*N* = 17,745,753)		*P*-value^b^
	
	*n*	%	%_wt_	*n*	%	
Gender						0.9980
Female	9,246	49.64	49.90	8,855,347	49.90	
Male	9,380	50.36	50.10	8,890,406	50.10	
Age, years						1.0000
12–17	3,598	19.32	8.21	1,456,619	8.21	
18–24	2,100	11.27	12.06	2,143,999	12.08	
25–29	1,373	7.37	9.04	1,600,050	9.02	
30–34	1,428	7.67	9.46	1,682,205	9.48	
35–39	1,609	8.64	11.46	2,028,089	11.43	
40–44	1,654	8.88	10.43	1,853,388	10.44	
45–49	1,618	8.69	10.18	1,804,240	10.17	
50–54	1,735	9.31	10.28	1,827,812	10.30	
55–59	1,891	10.15	10.09	1,779,179	10.03	
60–64	1,620	8.70	8.80	1,570,172	8.85	

^a^Source of data: Population by Sex and 5 Year Age Group for Counties and Cities at the End of Year in 2017. Taipei: Department of Household Registration, Ministry of the Interior, Executive Yuan. (https://www.ris.gov.tw/app/en/3910).

^b^*P*-value of chi-square goodness-of-fit test.

### Use and problematic use of substances

**Figure 1.**
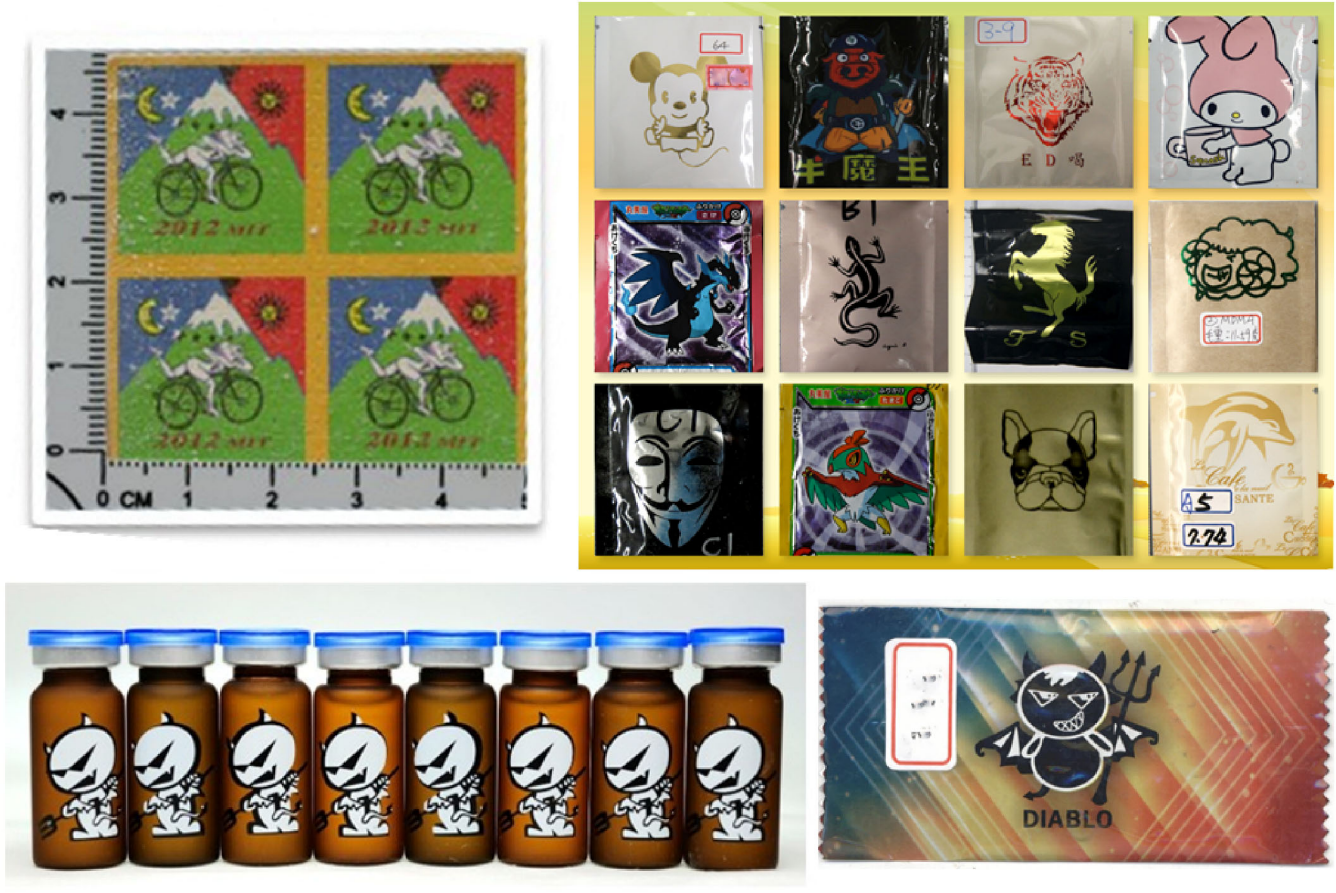
The colorful pictures of various illicit drug packets that had been seized by the law enforcement in Taiwan. More of such example pictures accompanied the question on prefixed drug in commodity packets on the screen in our tablet-based, computer-assisted self-interview in the 2018 National Survey of Substance Use in Taiwan. Source: Central Investigation Bureau, National Police Agency, Ministry of the Interior, Taiwan, as found on the Anti-Drug website (https://antidrug.moj.gov.tw/lp-1190-2.html) and used in accordance with the Open Data Statement (https://antidrug.moj.gov.tw/cp-116-6411-2.html) on that website.

Both the lifetime and past-year use prevalence of illicit drugs/inhalants and other psychoactive substances as well as scale-based problematic use of tobacco, alcohol, and drugs are displayed in Table 2. As the newly enlisted category of “prefixed drug in commodity packets” varied substantially in their forms, Figure 1 displays some pictures of such illicit drug packets seized by the law enforcement. Since the first reports of premixed drugs in commodity packets in Asia were only 3 years before this survey, we chose to focus on their lifetime use prevalence. Out of 18,626 participants, 262 (1.46%) had lifetime use of any illicit drug, with drugs in commodity packets (0.18%) being ranked the fifth most commonly used illicit drugs, lower than methamphetamine (0.42%), ketamine (0.40%), ecstasy (0.36%), and marijuana (0.32%) but higher than nitrous oxide (0.14%) and heroin (0.09%) (Table 1). In addition to ketamine, many other NPS drugs were used by the respondents, including GHB (0.07%), K2 (synthetic cannabinoids; 0.07%), Para-methoxymethamphetamine (0.01%), mephedrone (0.01%), and bath salts (synthetic cannabis; 0.01%). More information about individual illicit drugs/inhalants is provided in eTable 3.

**Table 2.** Prevalence of psychoactive substance use and problematic use among participants in the 2018 National Survey of Substance Use in Taiwan (*N* = 18,626)

Type of substance	Lifetime use			Past-year use		
	
	*n*	%_wt_	(SE)	*n*	%_wt_	(SE)
*Illicit drugs/inhalants use*						
Any illicit drug/inhalant	262	1.46	(0.11)	41	0.27	(0.05)
Methamphetamine	75	0.42	(0.06)	9	0.05	(0.02)
Ketamine	76	0.40	(0.06)	9	0.07	(0.03)
Ecstasy	67	0.36	(0.05)	6	0.02	(0.01)
Marijuana	60	0.32	(0.05)	10	0.03	(0.01)
Drugs in commodity packets	34	0.18	(0.04)	—	—	—
Nitrous oxide	19	0.14	(0.04)	5	0.04	(0.03)
Heroin	21	0.09	(0.02)	1	0.003	(0.003)

*Other psychoactive substance use*						
Alcohol	8,689	51.14	(0.49)	7,327	43.44	(0.49)
Cigarette	4,272	25.68	(0.44)	2,955	17.68	(0.38)
Areca nut	2,647	16.11	(0.37)	1,247	7.47	(0.26)
E-cigarette	750	4.18	(0.20)	285	1.56	(0.12)
Prescription sedatives – hypnotics	1,313	7.63	(0.26)	896	5.23	(0.22)
Nonmedical use	199	1.15	(0.11)	119	0.67	(0.08)
Prescription analgesics	1,079	6.38	(0.25)	720	4.41	(0.21)
Nonmedical use	329	1.91	(0.14)	213	1.33	(0.12)
Prescription stimulants	93	0.47	(0.06)	50	0.22	(0.04)
Nonmedical use	21	0.11	(0.03)	11	0.05	(0.02)

*Problematic use*						
Alcohol Use Disorder Identification Test						
Nondrinker				9,937	48.86	(0.49)
0–7				7,742	45.35	(0.49)
8–13				701	4.29	(0.21)
≥14				246	1.50	(0.12)
Fagerström Test for Nicotine Dependence						
Nonsmoker				14,354	74.32	(0.44)
0–3				2,695	16.40	(0.38)
≥4				1,577	9.28	(0.28)
Drug Abuse Screening Test-20						
Drug nonuser				16,252	86.34	(0.34)
0–5				2,250	12.99	(0.34)
≥6				124	0.66	(0.07)

%_wt_, weighted prevalence; SE, standard error.

### Formats of the commodity and lifetime use of other drugs

Among the 34 persons who reported having lifetime use of drugs in commodity packets, the various drug formats and their lifetime use of other drugs are displayed in Table 3. Ten different formats of drugs in commodity packets were endorsed, with the five most common types being instant coffee packets, instant milk tea packets, candy packets, snack packets, and jellies. Regarding the awareness of the contents in the packets, 11 persons (%_wt_ = 34.7%) reported that they had no idea of what drugs they were using. Regarding the lifetime use of other drugs among persons who used drug packets, 10 persons (%_wt_ = 18.3%) did not use any other illicit drugs, another 10 persons (%_wt_ = 32.9%) used one category of other illicit drugs/inhalants (mainly ketamine), and the remaining 14 persons (%_wt_ = 48.7%) used two or more categories of other illicit drugs/inhalants (mainly ketamine and ecstasy, with or without other drugs). Regarding treatment-seeking experience, a weighted proportion of 22% of persons with lifetime use of drugs in commodity packets received treatment for psychoactive drug use in the past year. For comparison, the corresponding figure was 5% for persons who exclusively used other illicit drugs.

**Table 3.** Formats of the commodity laced with illicit drugs and lifetime use of other drugs among persons with lifetime use of drugs in commodity packets (*N* = 34) in the 2018 National Survey of Substance Use in Taiwan

Drugs in commodity packets	*n*	Raw %	%_wt_^a^	(SE)
*Formats of the commodity laced with illicit drugs*				
Instant coffee packets	19	55.9	54.9	(4.2)
Instant milk tea packets	9	26.5	17.6	(5.0)
Candy packets	6	17.6	18.8	(0.4)
Snack packets	5	14.7	16.7	(2.2)
Drinks	1	2.9	3.0	(0.1)
Jellies	3	8.8	8.0	(4.6)
Little devils	2	5.9	9.0	(4.5)
Rainbow cigarettes	2	5.9	7.2	(0.1)
Other	1	2.9	7.3	(0.1)
*Awareness of contents* ^b^				
Unaware	11	34.4	34.7	(5.9)
Somewhat or fully aware	21	65.6	65.3	(5.9)
*Lifetime use of other drugs*				
Drug in commodity packets only	10	29.4	18.4	(4.8)
Drug in commodity packets + 1 other illicit drug/inhalant	10	29.4	32.9	(6.5)
Ketamine	4	11.8	9.3	(4.6)
N_2_O	2	5.9	11.4	(0.2)
Marijuana	2	5.9	6.0	(1.4)
Erimine	1	2.9	3.9	(3.9)
Methamphetamine	1	2.9	2.4	(2.4)
Drug in commodity packets + 2 other illicit drugs/inhalants	14	41.2	48.7	(6.1)
Ketamine + Ecstasy (+ other illicit drugs/inhalants)	11	32.4	39.7	(4.7)
Marijuana + Heroin (+ other illicit drugs/inhalants)	2	5.9	4.9	(3.5)
LSD + N_2_O	1	2.9	4.2	(0.9)
*Receiving treatment for psychoactive drug use in past year* ^c^				
Yes	8	24.2	22.0	(4.5)

%_wt_, weighted prevalence; LSD, lysergic acid diethylamide; N_2_O, nitrous oxide; SE, standard error.

^a^Weighted proportion, which was estimated using the sample weight of each person who reported having lifetime use of any drugs in commodity packets, with the total weight of the 34 persons being 33.6.

^b^Data were missing for 2 persons.

^c^Data were missing for 1 person.

### Sociodemographic correlates of the use of drugs in commodity packets

By dividing drug use into three groups (no drug use, drug in commodity packets use, and exclusive other drug use), we conducted multivariable multinomial logistic regressions of drug use on a variety of sociodemographic variables (Table 4). Compared to no drug use, the sociodemographic correlates for drug use in commodity packets included a higher risk associated with being aged 18–34 or 35–44 versus 45–64 years old and with being divorced or widowed versus being married. Meanwhile, the correlates for exclusive other drug use included a higher risk associated with being male, being aged 18–34 or 35–44 versus 45–64 years old, and having attained a lower educational level but a lower risk associated with being aged 12–17 years old. One notable difference in these sociodemographic correlates is the lack of a gender difference in drug use in commodity packets but not in the exclusive use of other drugs.

**Table 4.** Sociodemographic correlates of the use of drugs in commodity packets and other illicit drugs/inhalants among participants in the 2018 National Survey of Substance Use (*N* = 18,626)

Variables	*N*	No drug use		Drug in commodity packets use				Exclusive other drug use			
		
		*n*	%_wt_	*n*	%_wt_	aOR^a^	(95% CI)	*n*	%_wt_	aOR^a^	(95% CI)
Overall	18,626	18,364	98.54	34	0.18			228	1.28		
Gender											
Female	9,246	9,180	99.23	12	0.17	Ref		54	0.60	Ref	
Male	9,380	9,184	97.85	22	0.19	1.14	(0.47–2.73)	174	1.96	**3.61**	**(2.40–5.43)** ^*^
Age group, years											
45–64	6,864	6,796	99.13	6	0.06	Ref		62	0.81	Ref	
35–44	3,263	3,180	97.35	9	0.29	**8.36**	**(2.05–34.14)** ^*^	74	2.37	**4.10**	**(2.42–6.96)** ^*^
18–34	4,901	4,809	98.40	15	0.28	**7.11**	**(1.16–43.60)** ^*^	77	1.32	**2.39**	**(1.35–4.25)** ^*^
12–17	3,598	3,579	99.40	4	0.10	0.78	(0.09–6.47)	15	0.49	**0.33**	**(0.15–0.72)** ^*^
Urbanicity											
Urban	2,674	2,627	98.39	2	0.08	Ref		45	1.53	Ref	
Suburban	12,638	12,471	98.56	26	0.20	2.25	(0.49–10.42)	141	1.24	0.75	(0.50–1.12)
Rural	3,314	3,266	98.59	6	0.19	1.93	(0.33–11.25)	42	1.23	0.63	(0.38–1.06)
Marital status											
Married	8,479	8,380	98.77	8	0.07	Ref		91	1.15	Ref	
Divorced or widowed	1,216	1,185	97.70	8	0.57	**8.30**	**(2.58–26.66)** ^*^	23	1.73	1.51	(0.82–2.79)
Single	8,931	8,799	98.38	18	0.25	2.30	(0.55–9.52)	114	1.37	1.14	(0.76–1.73)
Educational attainment											
≥College	7,026	6,949	98.91	10	0.15	Ref		67	0.94	Ref	
Senior high school	5,184	5,079	98.17	15	0.22	1.56	(0.53–4.61)	90	1.61	**2.11**	**(1.42–3.14)** ^*^
≤Junior high school^b^	6,416	6,336	98.33	9	0.18	2.44	(0.38–15.92)	71	1.49	**4.02**	**(2.32–6.97)** ^*^
Occupation^c^											
Group I	6,323	6,212	98.47	10	0.13	Ref		101	1.41	Ref	
Group II	2,841	2,790	98.18	7	0.14	0.89	(0.24–3.27)	44	1.67	1.33	(0.85–2.07)
Group III	1,397	1,372	98.46	4	0.26	1.41	(0.32–6.34)	21	1.28	0.59	(0.33–1.06)
Group IV	8,065	7,990	98.82	13	0.24	2.05	(0.60–6.98)	62	0.95	0.97	(0.60–1.56)

%_wt_, weighted prevalence; aOR, adjusted odds ratio; CI, confidence interval; *N*, number of people in the stratum; *n*, number of people reporting the event.

^a^Adjusted for all the variables in this table by entering them as covariates in the multinomial logistic regression analysis.

^b^Including junior high school, elementary school, and not educated or unknown.

^c^Occupations: group I = other occupations; group II = service and sales workers; group III = plant and machine operators and assemblers or elementary laborers; group IV = unemployed or retired.

To explore the higher odds of being divorced or widowed with drugs in commodity packets use, we examined their self-reported relationship with family members in one DAST item. In terms of having problems with parents or partners over the drug use, people with use of drugs in commodity packets had a higher prevalence (*n* = 12, %_wt_ = 40.3%) than those with use of exclusive other drugs (*n* = 37, %_wt_ = 20.0%, *P* = 0.033). Next, to explore the higher odds of age group of 18–34/35–44 years old with drugs in commodity packets use, we found the proportion of being divorced or windowed in these two age groups was higher in females (1.9% and 8.0%) than in males (1.2% and 5.5%). More details are provided in eTable 8.

### Associations of other substance use with drug use in commodity packets

Similar multinomial logistic regression analyses of drug use on the use of other substances were conducted with adjustment for sociodemographic characteristics, including gender, age, urbanicity, marital status, educational level, and occupation (Table 5). Each of other substances was put in the model as independent variable separately. Compared to no drug use, an increased risk of exclusively using other illicit drugs was associated with alcohol use and a low-risk use of alcohol (AUDIT scores of 0–7), whereas the risk of drug use in commodity packets was not significantly increased with these two types of alcohol use. In the case of the use of substances other than alcohol, the pattern of significant correlations was similar between drug use in commodity packets and exclusive illicit drug use.

**Table 5.** Associations between the use of other substance or problematic use and the use of drugs in commodity packets and other illicit drugs/inhalants among participants in the 2018 National Survey of Substance Use (*N* = 18,626)

Variable	*N*	No drug use		Drug in commodity packets use				Exclusive other drug use			
		
		*n*	%_wt_	*n*	%_wt_	aOR^a^	(95% CI)	*n*	%_wt_	aOR^a^	(95% CI)
*Use of other substance*											
Alcohol use											
No	9,937	9,893	99.46	10	0.13	Ref		34	0.41	**Ref**	
Yes	8,689	8,471	97.66	24	0.23	1.73	(0.65–4.59)	194	2.11	**4.57**	**(2.75–7.58)** ^*^
Cigarette use											
No	14,354	14,299	99.62	10	0.08	Ref		45	0.31	**Ref**	
Yes	4,272	4,065	95.41	24	0.48	**10.13**	**(3.20–32.08)** ^*^	183	4.11	**12.90**	**(8.00–20.81)** ^*^
Areca nut use											
No	15,979	15,884	99.41	18	0.14	Ref		77	0.46	**Ref**	
Yes	2,647	2,480	94.00	16	0.42	**3.62**	**(1.26–10.40)** ^*^	151	5.58	**12.03**	**(7.94–18.23)** ^*^
E-cigarette use											
No	17,876	17,694	99.00	24	0.14	Ref		158	0.86	**Ref**	
Yes	750	670	87.98	10	1.01	**6.65**	**(2.46–17.95)** ^*^	70	11.01	**10.89**	**(7.05–16.81)** ^*^
Prescription sedatives – hypnotics use											
No	17,313	17,124	98.95	21	0.10	Ref		168	0.95	**Ref**	
Yes	1,313	1,240	93.55	13	1.14	**15.09**	**(6.47–35.15)** ^*^	60	5.31	**7.14**	**(4.83–10.55)** ^*^
Prescription analgesics use											
No	17,547	17,338	98.73	23	0.13	Ref		186	1.13	**Ref**	
Yes	1,079	1,026	95.63	11	0.86	**5.70**	**(2.20–14.80)** ^*^	42	3.51	**2.82**	**(1.80–4.42)** ^*^
Prescription stimulants use											
No	18,533	18,288	98.62	30	0.16	Ref		215	1.22	**Ref**	
Yes	93	76	81.02	4	3.97	**21.37**	**(5.37–85.06)** ^*^	13	15.01	**12.20**	**(5.60–26.57)** ^*^
*Problematic use*											
Alcohol Use Disorder Identification Test											
Nondrinker	9,937	9,893	99.46	10	0.13	Ref		34	0.41	**Ref**	
0–7	7,742	7,603	98.31	13	0.13	0.98	(0.34–2.82)	126	1.56	**3.59**	**(2.13–6.05)** ^*^
≥8	947	868	92.53	11	1.02	**7.65**	**(1.81–32.38)** ^*^	68	6.45	**10.98**	**(5.89–20.44)** ^*^
Fagerström Test for Nicotine Dependence											
Nonsmoker	14,354	14,299	99.62	10	0.08	Ref		45	0.31	Ref	
0–3	2,695	2,602	96.54	10	0.40	**8.76**	**(2.40–31.89)** ^*^	83	3.06	**10.22**	**(6.08–17.16)** ^*^
≥4	1,577	1,463	93.40	14	0.63	**13.76**	**(3.93–48.20)** ^*^	100	5.97	**19.89**	**(11.75–33.67)** ^*^

%_wt_, weighted prevalence; aOR, adjusted odds ratio; CI, confidence interval; *N*, number of people in the stratum; *n*, number of people reporting the event.

^a^For each substance use/problematic use variable, the adjusted odds ratio was obtained by controlling for all sociodemographic variables listed in Table 4, but not for the remaining substance use/problematic use variables in this table, in the model.

Since the frequency of outcome for the multinomial logistic regression was small in Table 4 and Table 5, we then performed post-hoc power analyses for these analyses. On the basis of observed ORs, the post-hoc power would be low-to-moderate if the OR for use of drug in commodity packets was around 2 to 3 (more details are provided in eTable 6 for sociodemographic correlates and eTable 7 for other substance use, and the relationship of OR and the standard error of log OR with the power of the test is depicted in eFigure 1).

### Gender differences in subgroups

**Figure 2.**
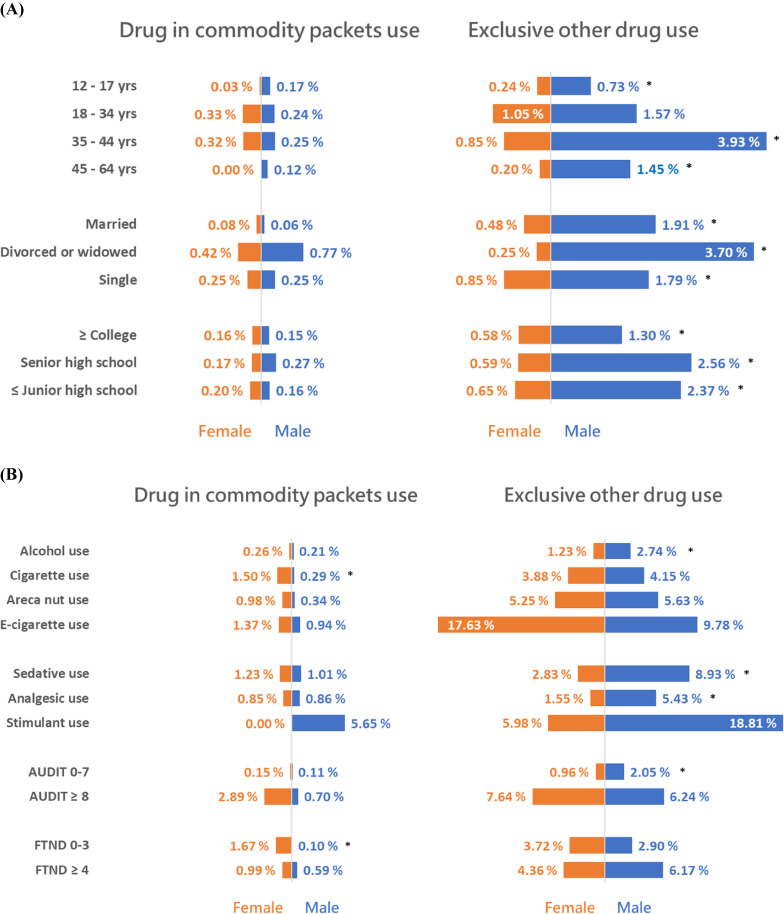
Gender-specific lifetime prevalence of the use of drugs in commodity packets and exclusive use of other drugs among participants in the 2018 National Survey of Substance Use in Taiwan by (**A**) sociodemographic subgroups and (**B**) other substance use groups. Asterisks (^*^) indicate significant differences in gender-specific prevalence estimates using the χ^2^ test.

We then examined whether gender convergence existed across subgroups of other sociodemographic and substance use variables. As displayed in Figure 2A, there were no significant gender differences for all the subgroups in age, marital status, or educational level in the use of drugs in commodity packets (numerical data in eTable 4). For all the individual age groups except 18–34 years, there were significant gender differences in the exclusive use of other drugs. Regarding the subgroup of other substance use (Figure 2B), there were two patterns of discordant gender differences (numerical data in eTable 5). The first pattern, which indicated excessive risk in females for drug use of commodity packets but no gender difference in the exclusive use of other drugs, was observed in the subgroups of cigarette use and low level of nicotine dependence (FTND scores of 0–3). The second pattern, which indicated no gender difference in the use of drugs in commodity packets but excessive risk in males for the exclusive use of other drugs, was observed in the subgroups of alcohol use, prescription sedative use, prescription analgesic use, and AUDIT scores of 0–7.

## DISCUSSION

Based on the 2018 national survey in Taiwan, the use of drugs in commodity packets had a lifetime prevalence of 0.18%, which was ranked as the fifth most commonly used illicit drug. Ten different formats of drugs in commodity packets were consumed by users. Of those who consumed drugs in commodity packets, 34.7% were unaware of the contents of the packets, and 81.6% had lifetime use of other illicit drugs. The sociodemographic correlates for the use of drugs in commodity packets were different from those of exclusive illicit drug use, particularly concerning the lack of gender differences in the former category in the whole sample, as well as in the subgroups of various sociodemographic characteristics and other substance use. These findings highlight the growing importance of drugs in commodity packets and have implications for drug testing and interventions.

Compared to two previous studies in clinical settings, one study of six treatment-seeking persons who used drugs in instant coffee packets in 2017^10^ and the other of 60 patients visiting the emergency department with ingestion of drugs in instant coffee packets between 2015 and 2019,^11^ our findings demonstrate that this form of NPS has been spreading in the population and has become as a common way of administering illicit drugs that deserves more attention.

This study further revealed a diversity in the formats of drug packets consumed by the respondents, and approximately one-third of the persons who used drugs in commodity packets were not aware of the contents, which might result in overdose or severe side effects, such that they received treatment for psychoactive drug at a higher rate than individuals who exclusively used other illicit drugs. These characteristics of the use of drugs in commodity packets pose new challenges to public health. The categories of illicit drugs included in packets are mixed, and the method of packing these packets is widely varied. It is likely that drug makers modify their packing methods once circulating forms are seized or intercepted by law enforcement. This creates further difficulties in drug testing. In a recent study among patients visiting the emergency department between 2017 and 2018 in Taipei, 32.0% tested positive for 16 types of NPSs using liquid chromatography-mass spectrometry/mass spectrometry, and the detected NPSs included ketamine (21.7%), 4-methyl-a-ethylaminopentiophenone (6.4%), mephedrone (4.4%), and methylone (4.4%).^30^ It is apparent that local law enforcement centers are not equipped with the facilities to analyze the contents of seized drugs in commodity packets. This diversity in NPSs also poses a challenge for drug regulation. In a comparative study of South Korea, Japan, and Taiwan, it was recommended that timely and flexible legislative mechanisms are essential for early identification, surveillance, and comprehensive evaluation.^3^

In our analyses of the correlates of drug use, there was an equal distribution between males and females of drug use in commodity packets, which was different from the male predominance in the exclusive use of other illicit drugs. For comparison, one study of six patients recruited from either an outpatient clinic or emergency department in a psychiatric hospital had a balanced gender distribution,^10^ whereas another study of 60 patients visiting the emergency department at three general hospitals reported a higher proportion of males (78.3%) than females.^11^ Since there might be different reasons for individuals to seek treatment in a clinical setting, the gender distribution is difficult to explain. It is noteworthy that diminishing gender differences have also been reported in the use of other illicit drugs, such as cannabis among the Japanese general population,^15^ party drugs among Dutch university students,^17^ heroin and opioid analgesics in the United States over the past decade,^31^^,^^32^ and NPS-related deaths in the United Kingdom.^33^ Similar trends were also found in problematic alcohol use in Taiwan^34^ and other East Asian countries.^35^^,^^36^ Whether changes in gender roles were associated with the phenomenon of gender convergence in various substance uses warrants future investigation.

For persons who use drugs in commodity packets, they had another feature that was different from those who exclusively used other drugs: a marital status of divorced or widowed was associated with use of drug in commodity packets but not use of exclusive other drugs. Intriguingly, users of drug in commodity packets reported a higher prevalence of having problems with parents or partners over the drug use than those who exclusively used other drugs. Among female participants who were divorced or widowed, their use prevalence of drugs in commodity packets was even higher than that of exclusive other drug use. In addition, females in the age groups of 18–34/35–44 years had higher proportion of being divorced or windowed than males. Taken together, it warrants for future investigation on whether use of drugs in commodity packets is a cause or result of divorce among young adult females.

Moreover, neither lifetime use nor low-risk use of alcohol (AUDIT scores of 0–7) was associated with an increased risk using drugs commodity packets. In contrast, there were strong associations of all types of alcohol use with the use of club drugs reported in the 2014 national survey.^19^ This implies that the users of drugs in commodity packets were different from young partygoers seeking club drugs.

Our findings have implications for illicit drug detection and intervention. First, given that the contents of drugs in commodity packets evolve quickly, a more feasible and efficient method of drug testing is warranted to facilitate effective law enforcement. One possible solution is equipping regional drug test centers with high-throughput analytic platforms, as exemplified by an automated workflow for multiple-reaction monitoring-based method that can simultaneously determine a variety of NPS classes in urine samples.^37^ Second, since most NPSs remain classified as Schedule III or IV controlled drugs, users are required to take 4–8 hours of drug seminar. Hence, culturally appropriate interventions for users of drugs in commodity packets are important for helping eliminate the misuse of these illicit drugs.

This study has several limitations. First, although an overall response rate of 64.6% was relatively good compared with the declining response rate to surveys over the last 30 years,^38^ county-specific response rates varied substantially. Although these response rates were incorporated in the final weights, there might be residual nonresponse bias. Nevertheless, interviewers in this study were asked to make two additional tries on different days or time to reach an individual if he or she was absent at first visit to diminish potential nonresponse bias. For those areas with relatively low response rates, their prevalence of illicit drug use might be more likely to be underestimated. Second, the prevalence of illicit drug use was based on self-report, which tends to be lower than that based on urinalysis.^39^ Nevertheless, using CASI as the mode of administration has been found to have higher reporting rates of drug-use behavior compared with those of traditional self-administered questionnaire.^40^^,^^41^ Third, since the post hoc power for the multinomial logistic regression analyses indicate a low-to-moderate power if the OR for use of drug in commodity packets is just around 2 to 3 (eTable 6), some sociodemographic correlates for the use of drug in commodity packets might have not been detected in our analyses. Meanwhile, the lack of gender differences for the use of drugs in commodity packets in some sociodemographic subgroups (see Figure 2) might also be due to the small number of such users. Future investigation with more drug-packets users is warranted. Last, the generalizability of our findings might be limited to those residents who were accessible at households.

In conclusion, the 2018 survey revealed the spreading of drugs in commodity packet use in the population in Taiwan. Unlike the exclusive use of other illicit drugs, users of drugs in commodity packets did not show gender differences. Our findings have implications for a more feasible and efficient method of testing for drugs in commodity packets and a culturally appropriate intervention for the users of this type of drug.

## SUPPLEMENTARY MATERIAL

eTable 1. The number of randomly selected civilians aged 12 to 64 years and the number of respondents completing the computer-assisted self-interview in the 2018 National Survey of Substance Use in Taiwan.

eTable 2. Test-retest reliability between 1–4 weeks apart for selected variables on sociodemographic characteristics and psychoactive substances among 31 participants

eTable 3. Lifetime and past-year use prevalence of illicit drugs/inhalants among participants in the 2018 National Survey of Substance Use in Taiwan (N = 18,626).

eTable 4. Gender-specific lifetime prevalence of the use of drugs in commodity packets and exclusive use of other drugs among participants in 2018 National Survey of Substance Use in Taiwan (N = 18,626) by sociodemographic subgroups.

eTable 5. Gender-specific lifetime prevalence of the use of drugs in commodity packets and exclusive use of other drugs among participants in 2018 National Survey of Substance Use in Taiwan (N = 18,626) by other substance use groups.

eTable 6. Post-hoc power estimation for the tests of variable effect in multinomial logistic regression applied in Table 4 based on observed odds ratios.

eTable 7. Post-hoc power estimation for the tests of variable effect in multinomial logistic regressions applied in Table 5 based on observed odds ratios.

eTable 8. Distribution of marital status among participants in 2018 National Survey of Substance Use in Taiwan, by sex and age group (N = 18,626)

eFigure 1. Post-hoc power analysis for the *t*-test of variable subgroup effect in multinomial logistic regression applied in this study (N = 18,626, d.f. = 18,576). Since the sample size of the survey was final, we used odds ratio (OR) and the standard error (SE) of log OR to show their relationships with the power of the test.

## References

[1] Peacock A, Bruno R, Gisev N, . New psychoactive substances: challenges for drug surveillance, control, and public health responses. Lancet. 2019;394(10209):1668–1684. 10.1016/S0140-6736(19)32231-7 31668410

[2] Shafi A, Berry AJ, Sumnall H, Wood DM, Tracy DK. New psychoactive substances: a review and updates. Ther Adv Psychopharmacol. 2020;10:2045125320967197. 10.1177/2045125320967197 33414905 PMC7750892

[3] Feng LY, Li JH. New psychoactive substances in Taiwan: challenges and strategies. Curr Opin Psychiatry. 2020;33(4):306–311. 10.1097/YCO.0000000000000604 32167950

[4] Hsu S. Instant beverage powders are laced with drugs: FDA. Taipei Times on January 7, 2015. Taipei, Taiwan (http://www.taipeitimes.com/News/taiwan/archives/2015/01/07/2003608676): Taipei Times; 2015.

[5] Chua A. Three held with Ecstasy in premix coffee The Star Online on March 12, 2015. Selangor Darul Ehsan, Malaysia (https://www.thestar.com.my/news/nation/2015/03/12/dangerous-drugs-in-happy-water-three-held-with-ecstasy-in-premix-coffee): Star Media Group Berhad; 2015.

[6] Abouchedid R, Ho JH, Hudson S, . Acute toxicity associated with use of 5F-derivations of synthetic cannabinoid receptor agonists with analytical confirmation. J Med Toxicol. 2016;12(4):396–401. 10.1007/s13181-016-0571-7 27456262 PMC5135680

[7] Usui K, Fujita Y, Kamijo Y, Kokaji T, Funayama M. Identification of 5-fluoro ADB in human whole blood in four death cases. J Anal Toxicol. 2018;42(2):e21–e25. 10.1093/jat/bkx088 29186561

[8] Creppage KE, Yohannan J, Williams K, . The rapid escalation of Fentanyl in illicit drug evidence in Allegheny County, Pennsylvania, 2010–2016. Public Health Rep. 2018;133(2):142–146. 10.1177/0033354917753119 29389251 PMC5871138

[9] Kant A, Mong R, Tan HH. Accidental ingestion of a novel psychoactive substance: a case report. Cureus. 2020;12(10):e11185. 10.7759/cureus.11185 33269117 PMC7703709

[10] Chang HM, Tracy DK, Huang MC, Pan CH, Chen LY. Psychiatric profiles and clinical manifestations of cathinone users: case series of analytically confirmed cathinone use in Taiwan. J Addict Addictv Disord. 2019;6:23. 10.24966/AAD-7276/100023

[11] Chou HH, Hsieh CH, Chaou CH, . Synthetic cathinone poisoning from ingestion of drug-laced “instant coffee packets” in Taiwan. Hum Exp Toxicol. 2021;40(9):1403–1412. 10.1177/0960327121996043 33715482

[12] United Nations Office on Drugs and Crime. World Drug Report 2017. I. Executive Summary: Conslusions and Policy Implications. Vienna: United Nations Office on Drugs and Crime; 2017.

[13] Palamar JJ, Le A. Use of new and uncommon synthetic psychoactive drugs among a nationally representative sample in the United States, 2005–2017. Hum Psychopharmacol Clin Exp. 2019;34(2):e2690. 10.1002/hup.2690 30843283 PMC6534815

[14] European Monitoring Centre for Drugs and Drug Addiction. High-risk drug use and new psychoactive substances: Results from an EMCDDA trendspotter study. Luxembourg: Publications Office of the European Union; 2017.

[15] Shimane T, Qiu D, Wada K. [Current situation of cannabis use in Japan: based on data from the Nationwide General Population Survey on Drug Use in Japan 2017]. Yakugaku Zasshi. 2020;140(2):173–178. 32009041 10.1248/yakushi.19-00195-1

[16] United Nations Office on Drugs and Crime. World Drug Report 2022. 1. Executive Summary, Policy Implications. Vienna: United Nations Office on Drugs and Crime; 2022.

[17] Kunst LE, Gebhardt WA. Prevalence and psychosocial correlates of party-drug use and associated problems among university students in the Netherlands. Subst Use Misuse. 2018;53(12):2077–2088. 10.1080/10826084.2018.1455700 29668345

[18] Liu CY, Yunh-Tai, Chen YJ, . Incorporating development stratification of Taiwan townships into sampling design of large scale health interview survey. J Health Manag. 2006;4:1–22.

[19] Chen WJ, Wu SC, Tsay WI, . Differences in prevalence, socio-behavioral correlates, and psychosocial distress between club drug and hard drug use in Taiwan: results from the 2014 National Survey of Substance Use. Int J Drug Policy. 2017;48:99–107. 10.1016/j.drugpo.2017.07.003 28810160

[20] Department of Household Registration at Ministry of the Interior. Population by Sex and 5 Year Age Group for Counties and Cities at the End of Year in 2016. (https://www.ris.gov.tw/app/en/3910) Taipei: Ministry of the Interior; 2018.

[21] Directorate-General of Budget Accounting and Statistics at Executive Yuan. 2010 Census of Agriculture, Forestry, Fishery, and Animal Husbandry. Taipei: Directorate-General of Budget, Accounting and Statistics, Executive Yuan; 2014.

[22] Department of Statistics at Ministry of Health and Welfare. Medical Care Institution’s Status & Hospital’s Utilization, 2016 (https://dep.mohw.gov.tw/DOS/cp-5099-62027-113.html). Taipei: Department of Statistics, Ministry of Health and Welfare; 2018.

[23] Heatherton TF, Kozlowski LT, Frecker RC, Fagerström KO. The Fagerström Test for Nicotine Dependence: a revision of the Fagerström Tolerance Questionnaire. Br J Addict. 1991;86(9):1119–1127. 10.1111/j.1360-0443.1991.tb01879.x 1932883

[24] Huang CL, Lin HH, Wang HH. Evaluating screening performances of the Fagerstrom tolerance questionnaire, the Fagerstrom test for nicotine dependence and the heavy smoking index among Taiwanese male smokers. J Clin Nurs. 2008;17(7):884–890. 10.1111/j.1365-2702.2007.02054.x 18321287

[25] Babor TF, Higgins-Biddle JC, Saunders JB, Monteiro MG. AUDIT-the Alcohol Use Disorders Identification Test: Guidelines for use in primary health care. 2nd ed ed. Geneva: World Health Organization; 2001.

[26] Chen CH, Chen WJ, Cheng ATA. New approach to the validity of the Alcohol Use Disorders Identification Test: stratum-specific likelihood ratios analysis. Alcohol Clin Exp Res. 2005;29(4):602–608. 10.1097/01.ALC.0000159189.56671.EC 15834225

[27] Skinner HA. Assessment of Substance Abuse: Drug Abuse Screening Test (DAST). Encyclopedia of Drugs, Alcohol, and Addictive Behavior: Retrieved January 10, 2016 from Encyclopedia.com: http://www.encyclopedia.com/doc/1G2-3403100068.html; 2001.

[28] Cocco KM, Carey KB. Psychometric properties of the Drug Abuse Screening Test in psychiatric outpatients. Psychol Assess. 1998;10(4):408–414. 10.1037/1040-3590.10.4.408

[29] Yudko E, Lozhkina O, Fouts A. A comprehensive review of the psychometric properties of the Drug Abuse Screening Test. J Subst Abuse Treat. 2007;32(2):189–198. 10.1016/j.jsat.2006.08.002 17306727

[30] Weng TI, Chen LY, Chen JY, Chen PS, Hwa HL, Fang CC. Characteristics of analytically confirmed illicit substance-using patients in the Emergency Department. J Formos Med Assoc. 2020;119(12):1827–1834. 10.1016/j.jfma.2020.01.005 32037264

[31] Marsh JC, Park K, Lin YA, Bersamira C. Gender differences in trends for heroin use and nonmedical prescription opioid use, 2007–2014. J Subst Abuse Treat. 2018;87:79–85. 10.1016/j.jsat.2018.01.001 29433788 PMC9084392

[32] McHugh RK, Nguyen MD, Chartoff EH, Sugarman DE, Greenfield SF. Gender differences in the prevalence of heroin and opioid analgesic misuse in the United States, 2015–2019. Drug Alcohol Depend. 2021;227:108978. 10.1016/j.drugalcdep.2021.108978 34488078 PMC8516063

[33] Webb L, Shi X, Goodair C, Cheeta S. Trends in mortality from novel psychoactive substances as “legal highs”: gender differences in manner of death and implications for risk differences for women. Front Psychiatry. 2022;13:890840. 10.3389/fpsyt.2022.890840 35530022 PMC9069007

[34] Huang YC, Wu SC, Hsiao PC, . Men’s decrease and women’s increase in harmful alcohol use from the 2014 to 2018 national surveys in Taiwan: a harbinger for an emerging national trend in East Asia? Int J Drug Policy. 2022;99:103441. 10.1016/j.drugpo.2021.103441 34503897

[35] Osaki Y, Kinjo A, Higuchi S, . Prevalence and trends in alcohol dependence and alcohol use disorders in Japanese adults; results from periodical nationwide surveys. Alcohol Alcohol. 2016;51(4):465–473. 10.1093/alcalc/agw002 26873982

[36] Choe SA, Yoo S, JeKarl J, Kim KK. Recent trend and associated factors of harmful alcohol use based on age and gender in Korea. J Korean Med Sci. 2018;33(4):e23. 10.3346/jkms.2018.33.e23 29318790 PMC5760808

[37] Chen JY, Chen GY, Wang SY, Fang CC, Chen LY, Weng TI. Development of an analytical method to detect simultaneously 219 new psychoactive substances and 65 other substances in urine specimens using LC-QqQ MS/MS with CriticalPairFinder and TransitionFinder. Talanta. 2022;238(Pt 1):122979. 10.1016/j.talanta.2021.122979 34857319

[38] National Research Council. Nonresponse in Social Science Surveys: A Research Agenda. Washington, DC: The National Academies Press; 2013.

[39] Chen WJ, Fang CC, Shyu RS, Lin KC. Underreporting of illicit drug use by patients at emergency departments as revealed by two-tiered urinalysis. Addict Behav. 2006;31:2304–2308. 10.1016/j.addbeh.2006.02.015 16564643

[40] Turner CF, Ku L, Rogers SM, Lindberg LD, Pleck JH, Sonenstein FL. Adolescent sexual behavior, drug, use, and violence: increased reporting with computer survey technology. Science. 1998;280:867–873. 10.1126/science.280.5365.867 9572724

[41] Wang YC, Lee CM, Lew-Ting CY, Hsiao CK, Chen DR, Chen WJ. Survey of substance use among high school students in Taipei: web-based questionnaire versus paper-and-pencil questionnaire. J Adolesc Health. 2005;37:289–295. 10.1016/j.jadohealth.2005.03.017 16182139

