# Supplementary material for: The Use of Premixed Drugs in Commodity Packets in the Population: Prevalence and Correlates Revealed by the 2018 National Survey of Substance Use in Taiwan
Source: J Epidemiol. 2024 May 5;34(5):218–27. doi: 10.2188/jea.JE20220356 (PMC10999518; doi:10.2188/jea.JE20220356)
Supplement: Supplementary file 1 [file je-34-218-s001.pdf]

**eTables**

eTable 1. The number of randomly selected civilians aged 12 to 64 years and the number of respondents completing the computer-assisted self-interview in the 2018 National Survey of Substance Use in Taiwan.

eTable 2. Test-retest reliability between 1-4 weeks apart for selected variables on sociodemographic characteristics and psychoactive substances among 31 participants

eTable 3. Lifetime and past-year use prevalence of illicit drugs/inhalants among participants in the 2018 National Survey of Substance Use in Taiwan (N = 18,626).

eTable 4. Gender-specific lifetime prevalence of the use of drugs in commodity packets and exclusive use of other drugs among participants in 2018 National Survey of Substance Use in Taiwan (N = 18,626) by sociodemographic subgroups.

eTable 5. Gender-specific lifetime prevalence of the use of drugs in commodity packets and exclusive use of other drugs among participants in 2018 National Survey of Substance Use in Taiwan (N = 18,626) by other substance use groups.

eTable 6. Post-hoc power estimation for the tests of variable effect in multinomial logistic regression applied in Table 4 based on observed odds ratios.

eTable 7. Post-hoc power estimation for the tests of variable effect in multinomial logistic regressions applied in Table 5 based on observed odds ratios.

eTable 8. Distribution of marital status among participants in 2018 National Survey of Substance Use in Taiwan, by sex and age group (N = 18,626)

**eFigures**

**eFigure 1.** Post-hoc power analysis for the t-test of variable subgroup effect in multinomial logistic regression applied in this study (N = 18,626, d.f. = 18,576). Since the sample size of the survey was final, we used odds ratio (OR) and the standard error (SE) of log OR to show their relationships with the power of the test.

**eTable 1.** The number of randomly selected civilians aged 12 to 64 years and the number of respondents completing the computer-assisted self-interview in the 2018 National Survey of Substance Use in Taiwan

| Area | Number Selected | Number Completed | Completion rate |
|---|---|---|---|
| Total | 28,840 | 18,626 | 64.6% |
| North | 9,312 | 4,904 | 52.7% |
| Keelung City | 1,100 | 666 | 60.5% |
| Taipei City | 1,512 | 469 | 31.0% |
| New Taipei City | 1,704 | 1,090 | 64.0% |
| Taoyuan City | 1,520 | 812 | 53.4% |
| Hsinchu City | 1,100 | 906 | 82.4% |
| Hsinchu County | 1,176 | 363 | 30.9% |
| Miaoli County | 1,200 | 598 | 49.8% |
| Central | 7,460 | 4,461 | 59.8% |
| Taichung City | 2,656 | 1,358 | 51.1% |
| Changhua County | 1,344 | 879 | 65.4% |
| Nantou County | 1,184 | 719 | 60.7% |
| Yunlin County | 1,172 | 882 | 75.3% |
| Penghu County | 1,104 | 623 | 56.4% |
| South | 8,732 | 6,888 | 78.9% |
| Chiai City | 1,100 | 899 | 81.7% |
| Chiai County | 1,200 | 840 | 70.0% |
| Tainan City | 2,552 | 2,330 | 91.3% |
| Kaohsiung City | 2,664 | 1,872 | 70.3% |
| Pingtung County | 1,216 | 947 | 77.9% |
| East | 3,336 | 2,373 | 71.1% |
| Yilan County | 1,104 | 823 | 74.5% |
| Hualien County | 1,112 | 780 | 70.1% |
| Taitung County | 1,120 | 770 | 68.8% |

**eTable 2.** Test-retest reliability between 1-4 weeks apart for selected variables on sociodemographic characteristics and psychoactive substance use among 31 participants

| Variable | Consistent answers | % |
|---|---|---|
| Year-month of birth | 31 | 100.0 |
| Sex | 31 | 100.0 |
| Marital status | 31 | 100.0 |
| Education | 31 | 100.0 |
| Occupation | 30 | 96.8 |
| Tobacco | 29 | 93.5 |
| Alcohol | 29 | 93.5 |
| Areca nut | 29 | 93.5 |
| Sedatives | 29 | 93.5 |
| Analgesics | 31 | 100.0 |
| Stimulants | 30 | 96.8 |
| Packed drugs | 31 | 100.0 |
| Ecstasy | 31 | 100.0 |
| Ketamine | 28 | 90.3 |
| Marijuana | 28 | 90.3 |
| Gamma hydroxybutyrate (GHB) | 29 | 93.5 |
| Mephedrone | 31 | 100.0 |
| Bath salts (synthetic cannabis) | 31 | 100.0 |
| Khat (alkaloid cathinone) | 31 | 100.0 |
| K2 (synthetic cannabinoids) | 31 | 100.0 |
| Lysergic acid diethylamide (LSD) | 31 | 100.0 |
| Erimine (nimetazepam) | 29 | 93.5 |
| Para-methoxymethamphetamin (PMMA) | 31 | 100.0 |
| Psilocybin mushroom | 31 | 100.0 |
| Methamphetamine | 31 | 100.0 |
| Heroin | 31 | 100.0 |
| Cocaine | 31 | 100.0 |
| Flunitrazepam (FM2) | 30 | 96.8 |
| Phencyclidine (PCP) | 31 | 100.0 |
| Pentazozine | 31 | 100.0 |
| Methadone | 31 | 100.0 |
| 2C-B (4-bromo-2,5-dimethoxyphenethylamine) | 30 | 96.8 |
| 5-MeO-DIPT (5-Methoxy-*N*,*N*-diisopropyltryptamine) | 31 | 100.0 |
| $N_2O$ | 31 | 100.0 |
| Glue | 31 | 100.0 |
| Rush (isobutyl nitrite) | 29 | 93.5 |

**eTable 3.** Lifetime and past-year use prevalence of illicit drugs/inhalants among participants in the 2018 National Survey of Substance Use in Taiwan (N=18,626)

| | Lifetime | | | Past-year | | |
|---|---|---|---|---|---|---|
| Illicit drugs/inhalants | n | $\%_{wt}$ | (SE) | n | $\%_{wt}$ | (SE) |
| Schedule I controlled drugs | | | | | | |
| Heroin | 21 | 0.09 | (0.02) | 1 | 0.003 | (0.003) |
| Cocaine | 3 | 0.01 | (0.01) | 1 | 0.001 | (0.001) |
| Schedule II controlled drugs | | | | | | |
| Methamphetamine | 75 | 0.42 | (0.06) | 9 | 0.05 | (0.02) |
| Ecstasy | 67 | 0.36 | (0.05) | 6 | 0.02 | (0.01) |
| Marijuana | 60 | 0.32 | (0.05) | 10 | 0.03 | (0.01) |
| Gamma hydroxybutyrate (GHB) | 13 | 0.07 | (0.02) | 4 | 0.01 | (0.01) |
| K2 (synthetic cannabinoids) | 12 | 0.07 | (0.03) | 3 | 0.03 | (0.02) |
| Methadone | 4 | 0.02 | (0.01) | 2 | 0.01 | (0.01) |
| Magic mushroom | 2 | 0.02 | (0.01) | 0 | 0.00 | (0.00) |
| Lysergic acid diethylamide (LSD) | 4 | 0.01 | (0.01) | 3 | 0.01 | (0.005) |
| Bath salts (synthetic cannabis) | 4 | 0.01 | (0.01) | 3 | 0.01 | (0.004) |
| Khat (alkaloid cathinone) | 1 | 0.001 | (0.001) | 1 | 0.001 | (0.001) |
| Pentazocine | 1 | 0.001 | (0.001) | 1 | 0.001 | (0.001) |
| Phencyclidine (PCP) | 0 | 0.00 | (0.00) | 0 | 0.00 | (0.00) |
| Schedule III controlled drugs | | | | | | |
| Ketamine | 76 | 0.40 | (0.06) | 9 | 0.07 | (0.03) |
| Flunitrazepam (FM2) | 15 | 0.09 | (0.03) | 8 | 0.05 | (0.03) |
| Erimine (nimetazepam) | 15 | 0.08 | (0.03) | 4 | 0.02 | (0.01) |
| Para-methoxymethamphetamin (PMMA) | 5 | 0.01 | (0.01) | 1 | 0.001 | (0.001) |
| Mephedrone | 3 | 0.01 | (0.004) | 2 | 0.005 | (0.004) |
| 2C-B (4-bromo-2,5-dimethoxyphenethylamine) | 1 | 0.001 | (0.001) | 1 | 0.001 | (0.001) |
| Schedule IV controlled drugs | | | | | | |
| 5-MeO-DIPT(5-Methoxy-*N*,*N*-opropyltryptamine) | 2 | 0.01 | (0.01) | 2 | 0.01 | (0.01) |
| Inhalants | | | | | | |
| Nitrous oxide | 19 | 0.14 | (0.04) | 5 | 0.04 | (0.03) |
| Glue | 12 | 0.06 | (0.02) | 2 | 0.01 | (0.01) |
| Rush (isobutyl nitrite) | 12 | 0.08 | (0.03) | 7 | 0.04 | (0.02) |
| Drugs in commodity packets | 34 | 0.18 | (0.04) | - | - | - |
| **Any of the above** | **262** | **1.46** | **(0.11)** | **41** | **0.27** | **(0.05)** |

**eTable 4.** Gender-specific lifetime prevalence of the use of drugs in commodity packets and exclusive use of other drugs among participants in 2018 National Survey of Substance Use in Taiwan (N = 18,626) by sociodemographic subgroups

| | Female | | | | Male | | | | $\chi^2$ test |
|---|---|---|---|---|---|---|---|---|---|
| | N | n | $\%_{wt}$ | (SE) | N | n | $\%_{wt}$ | (SE) | p-value |
| *Drug in commodity packets use* | | | | | | | | | |
| Age group, years | | | | | | | | | |
| 12–17 | 1,699 | 1 | 0.03 | (0.03) | 1,899 | 3 | 0.17 | (0.11) | 0.1141 |
| 18–34 | 2,306 | 6 | 0.33 | (0.17) | 2,595 | 9 | 0.24 | (0.09) | 0.6051 |
| 35–44 | 1,668 | 5 | 0.32 | (0.16) | 1,595 | 4 | 0.25 | (0.13) | 0.7880 |
| 45–64 | 3,573 | 0 | 0.00 | (0.00) | 3,291 | 6 | 0.12 | (0.05) | NA |
| Marital status | | | | | | | | | |
| Married | 4,520 | 4 | 0.08 | (0.05) | 3,959 | 4 | 0.06 | (0.03) | 0.6529 |
| Divorced or widowed | 712 | 2 | 0.42 | (0.36) | 504 | 6 | 0.77 | (0.36) | 0.5037 |
| Single | 4,014 | 6 | 0.25 | (0.13) | 4,917 | 12 | 0.25 | (0.08) | 0.9563 |
| Educational attainment | | | | | | | | | |
| ≥College | 3,488 | 5 | 0.16 | (0.09) | 3,538 | 5 | 0.15 | (0.07) | 0.9253 |
| Senior high school | 2,563 | 4 | 0.17 | (0.11) | 2,621 | 11 | 0.27 | (0.09) | 0.4906 |
| ≤Junior high school | 3,195 | 3 | 0.20 | (0.13) | 3,221 | 6 | 0.16 | (0.08) | 0.8214 |
| *Exclusive other drug use* | | | | | | | | | |
| Age group, years | | | | | | | | | |
| 12–17 | 1,699 | 5 | 0.24 | (0.11) | 1,899 | 10 | 0.73 | (0.26) | **0.0470** |
| 18–34 | 2,306 | 27 | 1.05 | (0.26) | 2,595 | 50 | 1.57 | (0.26) | 0.1748 |
| 35–44 | 1,668 | 13 | 0.85 | (0.31) | 1,595 | 61 | 3.93 | (0.62) | **<0.0001** |
| 45–64 | 3,573 | 9 | 0.20 | (0.08) | 3,291 | 53 | 1.45 | (0.23) | **<0.0001** |
| Marital status | | | | | | | | | |
| Married | 4,520 | 24 | 0.48 | (0.14) | 3,959 | 67 | 1.91 | (0.28) | **<0.0001** |
| Divorced or widowed | 712 | 3 | 0.25 | (0.16) | 504 | 20 | 3.70 | (1.07) | **<0.0001** |
| Single | 4,014 | 27 | 0.85 | (0.21) | 4,917 | 87 | 1.79 | (0.22) | **0.0061** |
| Educational attainment | | | | | | | | | |
| ≥College | 3,488 | 17 | 0.58 | (0.17) | 3,538 | 50 | 1.30 | (0.21) | **0.0151** |
| Senior high school | 2,563 | 23 | 0.59 | (0.14) | 2,621 | 67 | 2.56 | (0.38) | **<0.0001** |
| ≤Junior high school | 3,195 | 14 | 0.65 | (0.25) | 3,221 | 57 | 2.37 | (0.40) | **0.0010** |

NA, not applicable; SE, standard error.

**eTable 5.** Gender-specific lifetime prevalence of the use of drugs in commodity packets and exclusive use of other drugs among participants in 2018 National Survey of Substance Use in Taiwan (N=18,626) by other substance use groups

| | Female | | | | Male | | | | $\chi^2$ test |
|---|---|---|---|---|---|---|---|---|---|
| | N | n | $\%_{wt}$ | (SE) | N | n | $\%_{wt}$ | (SE) | p-value |
| *Drug in commodity packets use* | | | | | | | | | |
| Alcohol use | 3,573 | 8 | 0.26 | (0.11) | 5,116 | 16 | 0.21 | (0.06) | 0.6809 |
| Cigarette use | 687 | 8 | 1.50 | (0.60) | 3,585 | 16 | 0.29 | (0.08) | **0.0002** |
| Areca nut use | 314 | 4 | 0.98 | (0.58) | 2,333 | 12 | 0.34 | (0.11) | 0.1028 |
| E-cigarette use | 132 | 2 | 1.37 | (1.24) | 618 | 8 | 0.94 | (0.37) | 0.6361 |
| Sedative use | 789 | 6 | 1.23 | (0.54) | 524 | 7 | 1.01 | (0.44) | 0.8268 |
| Nonmedical sedative use | 221 | 1 | 0.66 | (0.65) | 131 | 2 | 0.52 | (0.40) | 0.9146 |
| Analgesic use | 527 | 4 | 0.85 | (0.48) | 552 | 7 | 0.86 | (0.40) | 0.9416 |
| Nonmedical analgesic use | 200 | 2 | 0.76 | (0.69) | 223 | 2 | 0.18 | (0.13) | 0.1773 |
| Stimulant use | 21 | 0 | 0.00 | (0.00) | 72 | 4 | 5.65 | (3.18) | NA |
| Nonmedical stimulant use | 9 | 0 | 0.00 | (0.00) | 30 | 2 | 7.18 | (5.25) | NA |
| AUDIT | | | | | | | | | |
| 0–7 | 3,427 | 6 | 0.15 | (0.07) | 4,315 | 7 | 0.11 | (0.05) | 0.6397 |
| ≥8 | 146 | 2 | 2.89 | (2.15) | 801 | 9 | 0.70 | (0.28) | 0.0649 |
| FTND | | | | | | | | | |
| 0–3 | 517 | 6 | 1.67 | (0.76) | 2,178 | 4 | 0.10 | (0.05) | **<.0001** |
| ≥4 | 170 | 2 | 0.99 | (0.77) | 1,407 | 12 | 0.59 | (0.20) | 0.5535 |
| *Exclusive other drug use* | | | | | | | | | |
| Alcohol use | 3,573 | 44 | 1.23 | (0.24) | 5,116 | 150 | 2.74 | (0.27) | **0.0002** |
| Cigarette use | 687 | 32 | 3.88 | (0.82) | 3,585 | 151 | 4.15 | (0.41) | 0.8111 |
| Areca nut use | 314 | 18 | 5.25 | (1.52) | 2,333 | 133 | 5.63 | (0.59) | 0.8374 |
| E-cigarette use | 132 | 19 | 17.63 | (4.60) | 618 | 51 | 9.78 | (1.57) | 0.0549 |
| Sedative use | 789 | 17 | 2.83 | (0.86) | 524 | 43 | 8.93 | (1.56) | **0.0005** |
| Nonmedical sedative use | 221 | 8 | 4.91 | (1.95) | 131 | 16 | 14.02 | (3.72) | **0.0234** |
| Analgesic use | 527 | 9 | 1.55 | (0.84) | 552 | 33 | 5.43 | (1.13) | **0.0192** |
| Nonmedical analgesic use | 200 | 2 | 0.51 | (0.37) | 223 | 14 | 4.80 | (1.42) | **0.0005** |
| Stimulant use | 21 | 1 | 5.98 | (5.84) | 72 | 12 | 18.81 | (6.58) | 0.1959 |
| Nonmedical stimulant use | 9 | 1 | 11.91 | (11.48) | 30 | 7 | 15.99 | (6.56) | 0.7193 |
| AUDIT | | | | | | | | | |
| 0–7 | 3,427 | 29 | 0.96 | (0.24) | 4,315 | 97 | 2.05 | (0.25) | **0.0047** |
| ≥8 | 146 | 15 | 7.64 | (2.30) | 801 | 53 | 6.24 | (1.07) | 0.5169 |
| FTND | | | | | | | | | |
| 0–3 | 517 | 23 | 3.72 | (0.95) | 2,178 | 60 | 2.90 | (0.45) | 0.3774 |
| ≥4 | 170 | 9 | 4.36 | (1.58) | 1,407 | 91 | 6.17 | (0.78) | 0.3653 |

AUDIT, Alcohol Use Disorders Identification Test; FTND, Fagerström Test for Nicotine Dependence; NA, not applicable; SE, standard error.

**eTable 6.** Post-hoc power estimation for the tests of variable effect in multinomial logistic regression applied in Table 4 based on observed odds ratios

| | | | "Drug in commodity packets use" vs. "No drug use" | | | | "Exclusive other drug use" vs. "No drug use" | | | |
|---|---|---|---|---|---|---|---|---|---|---|
| Variable | Subgroup | d.f. | log(OR) | SE of log(OR) | t | Power | log(OR) | SE of log(OR) | t | Power |
| Gender | Female | | | | | | | | | |
| | Male | 18,576 | 0.13 | 0.45 | 0.28 | 5.9% | 1.28 | 0.21 | 6.15 | 100.0% |
| Age group, years | 45–64 | | | | | | | | | |
| | 35–44 | 18,576 | 2.12 | 0.72 | 2.96 | 84.1% | 1.41 | 0.27 | 5.24 | 99.9% |
| | 18–34 | 18,576 | 1.96 | 0.92 | 2.12 | 56.4% | 0.87 | 0.29 | 2.97 | 84.4% |
| | 12–17 | 18,576 | -0.25 | 1.08 | -0.23 | 5.6% | -1.11 | 0.40 | -2.77 | 79.0% |
| Urbanicity | Urban | | | | | | | | | |
| | Suburban | 18,576 | 0.81 | 0.78 | 1.04 | 17.9% | -0.29 | 0.21 | -1.41 | 29.3% |
| | Rural | 18,576 | 0.66 | 0.90 | 0.73 | 11.3% | -0.46 | 0.26 | -1.75 | 41.7% |
| Marital status | Married | | | | | | | | | |
| | Divorced or widowed | 18,576 | 2.12 | 0.60 | 3.55 | 94.4% | 0.41 | 0.31 | 1.33 | 26.5% |
| | Single | 18,576 | 0.83 | 0.73 | 1.15 | 20.9% | 0.13 | 0.21 | 0.63 | 9.7% |
| Educational attainment | ≥College | | | | | | | | | |
| | Senior high school | 18,576 | 0.44 | 0.55 | 0.80 | 12.7% | 0.75 | 0.20 | 3.66 | 95.6% |
| | ≤Junior high school | 18,576 | 0.89 | 0.96 | 0.93 | 15.5% | 1.39 | 0.28 | 4.95 | 99.9% |
| Occupation | Group I | | | | | | | | | |
| | Group II | 18,576 | -0.11 | 0.66 | -0.17 | 5.3% | 0.28 | 0.23 | 1.25 | 24.1% |
| | Group III | 18,576 | 0.35 | 0.77 | 0.45 | 7.4% | -0.53 | 0.30 | -1.75 | 41.9% |
| | Group IV | 18,576 | 0.72 | 0.62 | 1.15 | 21.1% | -0.03 | 0.24 | -0.13 | 5.2% |

d.f., degrees of freedom; OR, odds ratio; SE, standard error.

**eTable 7.** Post-hoc power estimation for the tests of variable effect in multinomial logistic regressions applied in Table 5 based on observed odds ratios

| | | | "Drug in commodity packets use" vs. "No drug use" | | | | "Exclusive other drug use" vs. "No drug use" | | | |
|---|---|---|---|---|---|---|---|---|---|---|
| Variable | Subgroup | d.f. | log(OR) | SE of log(OR) | t | Power | log(OR) | SE of log(OR) | t | Power |
| **Use of other substance** | | | | | | | | | | |
| Alcohol use | No | | | | | | | | | |
| | Yes | 18,576 | 0.55 | 0.50 | 1.09 | 19.4% | 1.52 | 0.26 | 5.88 | 100.0% |
| Cigarette use | No | | | | | | | | | |
| | Yes | 18,576 | 2.32 | 0.59 | 3.94 | 97.6% | 2.56 | 0.24 | 10.49 | 100.0% |
| Areca nut use | No | | | | | | | | | |
| | Yes | 18,576 | 1.29 | 0.54 | 2.39 | 66.6% | 2.49 | 0.21 | 11.73 | 100.0% |
| E-cigarette use | No | | | | | | | | | |
| | Yes | 18,576 | 1.89 | 0.51 | 3.74 | 96.2% | 2.39 | 0.22 | 10.77 | 100.0% |
| Prescription sedatives - hypnotics use | No | | | | | | | | | |
| | Yes | 18,576 | 2.71 | 0.43 | 6.29 | 100.0% | 1.97 | 0.20 | 9.85 | 100.0% |
| Prescription analgesics use | No | | | | | | | | | |
| | Yes | 18,576 | 1.74 | 0.49 | 3.58 | 94.7% | 1.04 | 0.23 | 4.54 | 99.5% |
| Prescription stimulants use | No | | | | | | | | | |
| | Yes | 18,576 | 3.06 | 0.70 | 4.35 | 99.1% | 2.50 | 0.40 | 6.30 | 100.0% |
| **Problematic use** | | | | | | | | | | |
| Alcohol Use Disorder Identification Test | Nondrinker | | | | | | | | | |
| | 0–7 | 18,576 | -0.02 | 0.54 | -0.03 | 5.0% | 1.28 | 0.27 | 4.78 | 99.8% |
| | ≥8 | 18,576 | 2.03 | 0.74 | 2.76 | 78.9% | 2.40 | 0.32 | 7.55 | 100.0% |
| Fagerström Test for Nicotine Dependence | Nonsmoker | | | | | | | | | |
| | 0–3 | 18,576 | 2.17 | 0.66 | 3.29 | 90.8% | 2.32 | 0.26 | 8.79 | 100.0% |
| | ≥4 | 18,576 | 2.62 | 0.64 | 4.10 | 98.4% | 2.99 | 0.27 | 11.14 | 100.0% |

d.f., degrees of freedom; OR, odds ratio; SE, standard error.

**eTable 8.** Distribution of marital status among participants in 2018 National Survey of Substance Use in Taiwan, by sex and age group (N=18,626)

| | 12–17 years (N=3,598) | | 18–34 years (N=4,901) | | 35–44 years (N=3,263) | | 45–64 years (N=6,864) | |
|---|---|---|---|---|---|---|---|---|
| Marital status | n | $\%_{wt}$ | n | $\%_{wt}$ | n | $\%_{wt}$ | n | $\%_{wt}$ |
| Overall | | | | | | | | |
| Married | 0 | 0.0 | 926 | 19.9 | 2,171 | 66.8 | 5,382 | 78.4 |
| Divorced or widowed | 0 | 0.0 | 75 | 1.6 | 248 | 6.8 | 893 | 12.8 |
| Single | 3,598 | 100.0 | 3,900 | 78.6 | 844 | 26.4 | 589 | 8.7 |
| Female | | | | | | | | |
| Married | 0 | 0.0 | 567 | 25.6 | 1,170 | 69.7 | 2,783 | 78.4 |
| Divorced or widowed | 0 | 0.0 | 38 | 1.9 | 143 | 8.0 | 531 | 14.1 |
| Single | 1,699 | 100.0 | 1,701 | 72.5 | 355 | 22.3 | 259 | 7.5 |
| Male | | | | | | | | |
| Married | 0 | 0.0 | 359 | 14.4 | 1,001 | 63.8 | 2,599 | 78.5 |
| Divorced or widowed | 0 | 0.0 | 37 | 1.3 | 105 | 5.5 | 362 | 11.5 |
| Single | 1,899 | 100.0 | 2,199 | 84.3 | 489 | 30.6 | 330 | 10.1 |

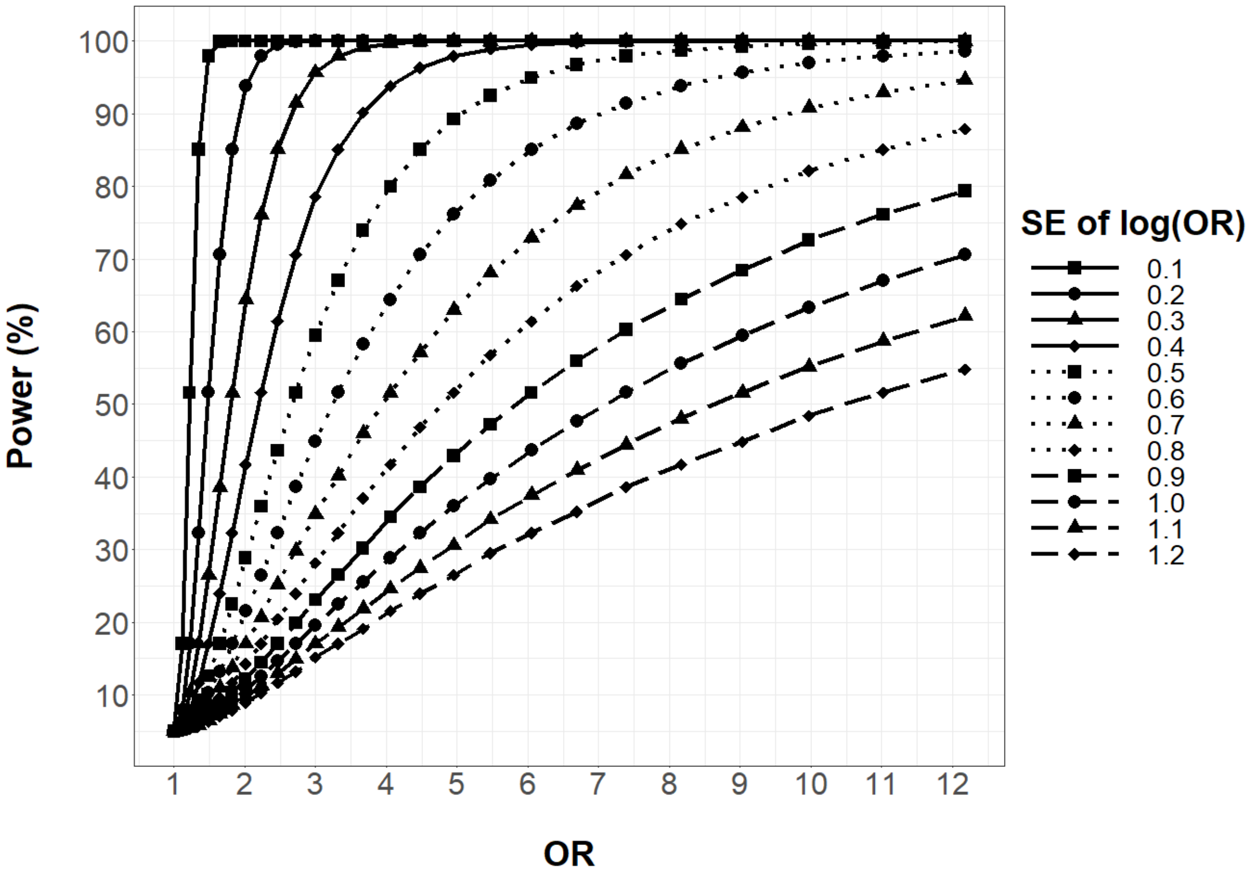


**eFigure 1.** Post-hoc power analysis for the t-test of variable subgroup effect in multinomial logistic regression applied in this study (N=18,626, d.f.=18,576). Since the sample size of the survey was final, we used odds ratio (OR) and the standard error (SE) of log OR to show their relationships with the power of the test.
